# Validation to Brazilian Portuguese of the Self-Care Inventory-revised for adults with type 2 diabetes

**DOI:** 10.20945/2359-3997000000213

**Published:** 2020-03-18

**Authors:** Gabriela H. Teló, Fernando de Quadros Iorra, Bruna S. Velho, Karen Sparrenberger, Beatriz D. Schaan

**Affiliations:** 1 Departamento de Medicina Interna Escola de Medicina Pontifícia Universidade Católica do Rio Grande do Sul Porto Alegre RS Brasil Departamento de Medicina Interna, Escola de Medicina da Pontifícia Universidade Católica do Rio Grande do Sul, Porto Alegre, RS, Brasil; 2 Departamento de Medicina Interna Universidade Federal do Rio Grande do Sul Porto Alegre RS Brasil Departamento de Medicina Interna, Universidade Federal do Rio Grande do Sul, Porto Alegre, RS, Brasil; 3 Serviço de Endocrinologia Hospital de Clínicas de Porto Alegre Porto Alegre RS Brasil Serviço de Endocrinologia, Hospital de Clínicas de Porto Alegre, Porto Alegre, RS, Brasil

**Keywords:** Validation, survey, adherence, type 2 diabetes

## Abstract

**Objective:**

The aim of this study was to cross-culturally adapt and validate the Brazilian Portuguese version of SCI-R to adults with type 2 diabetes.

**Materials and methods:**

The SCI-R is a 15-question survey that reflects how well patients with diabetes have adhered to treatment recommendations. A pretest (n = 40) was first performed to improve comprehension of the survey items. A final version was then self-administered in another 75 adults with type 2 diabetes to determine the survey’s reliability and validity according to its association with HbA1c. Finally, we conducted a test-retest reliability analysis over three weeks to stabilize the sample and determine intra-observer variability.

**Results:**

After the pretest phase, the final sample’s (N = 75) mean age was 59.9 ± 7.5 years and mean HbA1c level was 8.6 ± 1.5% (70 ± 16.4 mmol/mol). The initial Cronbach’s alpha was 0.61; however, further analysis showed that four items had low item correlation and were excluded from the final version, which increased the Cronbach’s alpha to 0.63. In predictive validity analysis, HbA1c levels correlated significantly with total survey scores (r = -0.38, P = 0.001). The intra-class correlation coefficient between baseline and three-week scores was 0.93, which indicates high reproducibility.

**Conclusions:**

The Brazilian Portuguese version of the SCI-R is a valid tool for measuring treatment adherence in adults with type 2 diabetes.

## INTRODUCTION

It is estimated that diabetes affects 14.3 million adults in Brazil, which represents 10.2% of the Brazilian population aged 20-79 years (1), ranking high among the health problems in this country (2). Despite all the evidence that glycemic control can be optimized and microvascular and macrovascular complications can be prevented (3,4), adherence to diabetes treatment has still been a challenge (5). In Brazil, most type 2 diabetes patients present poor glycemic control, which is strongly associated with poor adherence to diabetes treatment (6). Among those treated in the public healthcare system, the hemoglobin A1c (HbA1c) levels of less than 30% were within the American Diabetes Association target (6,7).

There are several key elements in diabetes management, including self-care education about medication, physical activity, nutrition and blood glucose monitoring (7,8). Considering that improved treatment adherence could optimize glycemic control (5,9,10), instruments that assess patient compliance are essential. However, cultural barriers have limited the available tools for Brazilian adults with type 2 diabetes.

Diabetes-specific instruments have high acceptance among patients and allow evaluation of specific aspects of the disease. The number of cross-culturally adapted or validated questionnaires for Brazilian culture is still insufficient for the national demand. The Self-Care Inventory – Revised (SCI-R) is a brief, psychometrically sound measure of adherence to recommended diabetes self-care behaviors in adults with diabetes (11). This survey has been previously validated in Brazil in a population of adults with type 1 diabetes but not type 2 diabetes (12). Currently, no Brazilian Portuguese survey has been validated to evaluate treatment adherence in both type 1 and type 2 diabetes. The aim of this study was to cross-culturally adapt and validate the Brazilian Portuguese version of the SCI-R in adults with type 2 diabetes.

## MATERIALS AND METHODS

### Participants and setting

This study was carried out from January to December 2016 in a diabetes outpatient clinic of the Hospital de Clínicas de Porto Alegre, a tertiary university hospital in Southern Brazil. Patients were selected by searching the hospital’s medical record database, and those who met the eligibility criteria were contacted by telephone and provided with information about the study. Patients were eligible if they were aged 18 and older, had been previously diagnosed with type 2 diabetes and had Brazilian cultural identity. Exclusion criteria consisted of any communication or understanding barrier, such as mental disorder or illiteracy. Such barriers were identified based on self, family or medical reports. The local institutional review board approved this study protocol, and all participants provided written informed consent.

### Instrument and procedures

The SCI-R is a self-administered 15-question survey that uses a 5-point Likert scale to reflect how well respondents have followed diabetes treatment recommendations over the previous 1-2 months (i.e., 1 = “never” to 5 = “always”). Higher scores indicate better adherence. In Brazil, this measure has been recently validated in adults with type 1 diabetes, showing acceptable internal reliability and correlation between glycemic control and total score (12).

Trained researchers collected the demographic and clinical data. Prior to application of the survey, the participants were asked about clinical data, such as medications and diabetes complications, which was confirmed through their electronic medical records. Some diabetes variables, such as age at diagnosis and diabetes duration were obtained in years, as were some demographics, such as education level. HbA1c measurements were obtained by high-performance liquid chromatography, either from medical records if collected in the month prior to taking the survey or, when not available, from a new blood sample.

Details of the transcultural adaptation of the original SCI-R have been described elsewhere (12), i.e. the translation, back-translation and revision strategies of the Portuguese version by a committee of specialists, including two endocrinologists and a linguistics specialist, among other researchers.

To enhance the instrument’s construct validity, the previously adapted version (12) was first applied to 40 patients (13) in a pretest to discover the respondents’ interpretation of each item. Based on this feedback, modifications were made in certain items to ensure easy comprehension. Upon the conclusion of the adaptation process, and based on a suggestion regarding validation sample size (13), another 75 patients were selected to answer the final version of SCI-R to determine the instrument’s reliability and validity according to association with HbA1c levels. Finally, we conducted a test-retest reliability analysis over three weeks in 25% of the sample (n = 20) to stabilize the sample and determine intra-observer variability.

### Statistical analyses

Internal consistency was assessed by calculating Cronbach’s alpha for total score and item-total score correlations. Values > 0.6 were considered acceptable. The impact of removing each item on the Cronbach’s alpha value was then assessed, and a low item-correlation could lead to the exclusion of a particular question. Pearson’s correlation coefficient was used to determine the predictive validity between total SCI-R scores and HbA1c levels. Intra-class correlation was used to evaluate intra-observer variability. The sample size was based on recommendations in the literature for survey validation studies (13).

## RESULTS

A total of 242 patients were enrolled in this study. Of these, 54 declined to participate, six were excluded due to cognitive or language impairment and 67 did not have a valid telephone contact or did not answer calls. The first 40 patients included were selected for the pretest phase, i.e. completing the survey and indicating comprehension difficulties in each question. This led to modifications in five items to facilitate comprehension (see supplementary Table 1). The main strategy in this process was adding parenthetical statements after nouns to provide additional information. For example, in item one, “finger glucose meter device” was added to the item “verify blood glucose level with a monitor”. After this step, the next 75 patients included were considered the final sample and completed the adjusted version of the survey. We found no significant differences in age, sex or diabetes duration between participants and decliners, or between the pretest and final samples.

Overall (N = 75), the mean age was 59.9 ± 7.5 years; 59% were women and 71% were Caucasian. The mean diabetes duration was 16.5 ± 8.6 years, and the mean HbA1c level was 8.7 ± 1.5% (72 ± 16.4 mmol/mol) (see Table 1). The SCI-R survey was self-administered and completed within 8-10 minutes. The initial Cronbach’s alpha was 0.61. The analysis showed that four items (checking ketones, modifying the medication dose, eating only the recommended amount of carbohydrates while hypoglycemic, and wearing a medical alert) had low-item correlation and were excluded from the final SCI-R version, which increased the Cronbach’s alpha to 0.63. In predictive validity analysis, HbA1c levels correlated significantly with total survey scores (r = -0.38, P = 0.001). Test-retest reliability was analyzed over three weeks using data from 25% of the sample (n = 20). The intra-class correlation coefficient between baseline and three-week scores was 0.93, which indicates high reproducibility.

**Table 1:** Participant demographics and clinical characteristics

Characteristics (N = 75)	Mean ± SD or %
Age (years)	59.9 ± 7.5
Sex (% female)	59
Ethnicity (% Caucasian)	71
Educational level (years of study)	7.6 ± 3.1
Weight status	-
Normal (%)	9
Overweight (%)	23
Obese (%)	68
Age at diabetes diagnosis (years)	43.3 ± 9.2
Diabetes duration (years)	16.5 ± 8.6
HbA1c (%)	8.7 ± 1.5
HbA1c (mmol/mol)	72 ± 16.4
Chronic diabetes complication (%)	79
Insulin use (%)	80

Data are mean ± standard deviation (SD) or %. HbA1c: hemoglobin A1c.

## DISCUSSION

Due to the increasing number of diabetes cases in Brazil, it has become necessary to develop specific instruments to help health professionals with the follow-up of these patients. We conducted a complementary adjustment process of the SCI-R survey, which had been previously cross-culturally adapted to Brazilian Portuguese and validated for type 1 diabetes (12). In this study, we evaluated the reliability and validity of this survey in Brazilian adults with type 2 diabetes. To our knowledge, this is the first survey validated in Brazil for evaluating treatment adherence in both type 1 and type 2 diabetes populations. The questionnaire presented satisfactory psychometric properties and is thus an acceptable instrument for measuring treatment adherence in the studied population.

Cronbach’s alpha is used to establish internal consistency, and results higher than 0.6 are considered acceptable when evaluating measurement instruments. We found, as in other validations of this same survey (12,14), a lower Cronbach’s alpha than the original SCI-R study (α = 0.87) (11). This difference could be partially explained by the fact that the original questionnaire was applied in both type 1 and type 2 diabetes patients. Its strict use in type 2 diabetes patients in this study led to the loss of four questions that were mainly related to type 1 diabetes treatment and, as a result, the final version included only 11 items. Furthermore, although easy to use, the survey is self-applied, which increases the probability of operational mistakes, such as misinterpreting instructions.

In the United Kingdom validation of the SCI-R survey for adults with type 2 diabetes (14), the authors evaluated how many questions could be excluded from the original survey without losing reliability, concluding that at least 11 questions should be maintained. Similar to our findings, this confirms the importance of retaining most items when adapting instruments to ensure reliability, despite the possibility of making an instrument more tiresome and less attractive.

In conclusion, the Brazilian Portuguese version of the SCI-R is an acceptable instrument for assessing treatment adherence in Brazilian adults with type 2 diabetes. The main purpose of this easy-to-use resource is to provide a general view of the patients’ self-care behaviors, serving as an accessory in clinical practice and a research instrument for measuring and interpreting individual compliance.

Ethics approval and consent to participate: this study was approved (15-0554) by the *Hospital de Clínicas de Porto Alegre* Institutional Ethics Committee, which is certified by the Office of Human Research Protections as an institutional review board. All respondents signed an informed consent form prior to participation in any study procedure.

## Supplementary Table 1

*Original Version*

INVENTÁRIO DE AUTOCUIDADO – DIABETES (SCI-R)

Este questionário avalia o que você realmente faz, e não o que você é recomendado a fazer.

Para cada pergunta abaixo, como você tem seguido o seu tratamento do diabetes nos últimos 1 a 2 meses?

**Table:**

	Nunca	Raramente	Às vezes	Geralmente	Sempre
1. Verifica a glicose no sangue com monitor	①①	②②	③③	④④	⑤⑤
2. Anota os resultados de glicose no sangue	①①	②②	③③	④④	⑤⑤
3. Verifica cetonas quando o nível de glicose está alto	①①	②②	③③	④④	⑤⑤
4. Usa a dose correta de insulina	①①	②②	③③	④④	⑤⑤
5. Usa a insulina na hora certa	①①	②②	③③	④④	⑤⑤
6. Come as porções corretas de comida	①①	②②	③③	④④	⑤⑤
7. Come as refeições e lanches na hora certa	①①	②②	③③	④④	⑤⑤
8. Anota o que come	①①	②②	③③	④④	⑤⑤
9. Lê os rótulos dos alimentos	①①	②②	③③	④④	⑤⑤
10. Trata a glicose baixa no sangue com somente a quantidade recomendada de carboidratos	①①	②②	③③	④④	⑤⑤
11. Carrega açúcar para, em caso de emergência, tratar a glicose baixa no sangue	①①	②②	③③	④④	⑤⑤
12. Comparece às consultas marcadas	①①	②②	③③	④④	⑤⑤
13. Faz exercícios	①①	②②	③③	④④	⑤⑤
14. Ajusta a dose de insulina baseado nos valores da glicose, comida e exercícios	①①	②②	③③	④④	⑤⑤

*Current Version*

INVENTÁRIO DE AUTOCUIDADO – DIABETES (SCI-R)

Este questionário avalia o que você realmente faz, e não o que você é recomendado a fazer.

Para cada pergunta abaixo, como você tem seguido o seu tratamento do diabetes nos últimos 1 a 2 meses?

**Table:**

	Nunca	Raramente	Às vezes	Geralmente	Sempre	
1. Verifica a glicose no sangue com monitor (aparelho de medir glicose no dedo)	①	②	③	④	⑤	
2. Anota os resultados de glicose no sangue quando verifica com o monitor	①	②	③	④	⑤	
3. Verifica cetonas no sangue ou na urina quando o nível de glicose está alto	①	②	③	④	⑤	
4. Usa a dose correta de insulina ou dos remédios para diabetes	①	②	③	④	⑤	Não usa insulina ou remédios para diabetes
5. Usa a insulina ou os remédios para diabetes na hora certa	①	②	③	④	⑤	Não usa insulina ou remédios para diabetes
6. Come as porções corretas de comida	①	②	③	④	⑤	
7. Come as refeições e lanches na hora certa	①	②	③	④	⑤	
8. Anota o que come	①	②	③	④	⑤	
9. Lê os rótulos dos alimentos	①	②	③	④	⑤	
10. Carrega carboidrato para, em caso de emergência, tratar a glicose baixa no sangue	①	②	③	④	⑤	
11. Quando a glicose no sangue está baixa, trata somente com a quantidade de carboidratos recomendada	①	②	③	④	⑤	Não teve glicose baixa no sangue
12. Comparece às consultas marcadas	①	②	③	④	⑤	
13. Carrega algum tipo de identificação que comprove o diabetes (por exemplo: cartão, pulseira, colar)	①	②	③	④	⑤	
14. Faz exercícios	①	②	③	④	⑤	
15. Você modifica a dose de insulina baseado nos valores da glicose, comida e/ou exercícios	①	②	③	④	⑤	Não usa insulina
